# Ginsenoside Rc, an Active Component of *Panax ginseng*, Alleviates Oxidative Stress-Induced Muscle Atrophy via Improvement of Mitochondrial Biogenesis

**DOI:** 10.3390/antiox12081576

**Published:** 2023-08-07

**Authors:** Aeyung Kim, Sang-Min Park, No Soo Kim, Haeseung Lee

**Affiliations:** 1 Korean Medicine (KM) Application Center, Korea Institute of Oriental Medicine, Daegu 41062, Republic of Korea; 2 College of Pharmacy, Chungnam National University, Daejeon 34134, Republic of Korea; smpark@cnu.ac.kr; 3 KM Convergence Research Division, Korea Institute of Oriental Medicine, Daejeon 34054, Republic of Korea; nosookim@kiom.re.kr; 4 College of Pharmacy and Research Institute for Drug Development, Pusan National University, Busan 46241, Republic of Korea

**Keywords:** ginsenoside Rc, skeletal muscle, oxidative stress, mitochondrial biogenesis, muscle atrophy

## Abstract

Loss of skeletal muscle mass and function has detrimental effects on quality of life, morbidity, and mortality, and is particularly relevant in aging societies. The enhancement of mitochondrial function has shown promise in promoting muscle differentiation and function. Ginsenoside Rc (gRc), a major component of ginseng, has various pharmacological activities; however, its effect on muscle loss remains poorly explored. In this study, we examined the effects of gRc on the hydrogen peroxide (H_2_O_2_)-induced reduction of cell viability in C2C12 myoblasts and myotubes and H_2_O_2_-induced myotube degradation. In addition, we investigated the effects of gRc on the production of intracellular reactive oxygen species (ROS) and mitochondrial superoxide, ATP generation, and peroxisome proliferator-activated receptor-gamma co-activator 1α (PGC-1α) activity in myoblasts and myotubes under H_2_O_2_ treatment. Furthermore, to elucidate the mechanism of action of gRc, we conducted a transcriptome analysis of myotubes treated with or without gRc under H_2_O_2_ treatment. gRc effectively suppressed H_2_O_2_-induced cytotoxicity, intracellular ROS, and mitochondrial superoxide production, restored PGC-1α promoter activity, and increased ATP synthesis. Moreover, gRc significantly affected the expression levels of genes involved in maintaining mitochondrial mass and biogenesis, while downregulating genes associated with muscle degradation in C2C12 myotubes under oxidative stress. We provide compelling evidence supporting the potential of gRc as a promising treatment for muscle loss and weakness. Further investigations of the pharmacological effects of gRc under various pathological conditions of muscle loss will contribute to the clinical development of gRc as a therapeutic intervention.

## 1. Introduction

Skeletal muscle accounts for approximately 40–50% of the human body and plays a pivotal role in maintaining the motion, posture, and movement of various organs. Skeletal muscle mass decreases by approximately 1% per year after 30 years of age and rapidly decreases after 65 years of age [1,2]. Decreased muscle mass causes decreased exercise capacity, falls, osteoporosis, and fractures, which are closely related to a decreased quality of life, increased hospitalization, and increased mortality. In addition, reduced physical activity and total energy expenditure due to decreased muscle mass and function significantly increases weight gain, decreases lung function, and increases the prevalence of cardiovascular and metabolic diseases [3,4,5]. These diseases promote muscle loss through feedback mechanisms. Muscle loss can be caused by damage and dysfunction of mitochondria, which are organelles within muscle cells [6]. Chronic diseases such as pulmonary tuberculosis and diabetes, malignant wasting diseases such as cancer cachexia, long-term administration of drugs such as glucocorticoids and statins, oxidative stress, disuse due to surgery and long-term bed rest, and aging-related hormonal changes are the main causes of impaired mitochondrial function in muscles [6,7]. Therefore, to improve muscle mass and function, it is important to enhance mitochondrial quantity and function by increasing mitochondrial biogenesis and regulating mitochondrial dynamics and mitophagy.

Adequate levels of reactive oxygen species (ROS) are required to regulate various signaling pathways; however, excessive and sustained ROS production in cells can trigger an oxidative damage response [8]. As mitochondria are a major source and the most vulnerable targets of ROS, inappropriate ROS accumulation reduces mitochondrial membrane potential and activates the caspase cascade, leading to mitochondrial dysfunction-induced apoptosis [9]. Similar to many other cell types, oxidative stress in skeletal muscle can trigger mitochondrial DNA damage and lead to defects in myogenesis and muscle regeneration. Additionally, oxidative stress plays an important role in the pathophysiology of various muscle diseases including age-related loss of muscle quantity (sarcopenia) and strength (dynapenia), muscular dystrophy, and myopathies [10,11]. The balance between ROS generation and antioxidant defenses is critical for maintaining muscle redox homeostasis [12]. Therefore, various attempts have been made to strengthen muscle cells by removing excessive ROS using various antioxidants and preventing apoptosis and protein degradation in the skeletal muscle.

Ginseng root (*Panax ginseng* Meyer) is one of the most widely used herbal medicines and has been prescribed for over 2000 years in Asian countries, including Korea, China, and Japan [13]. Ginseng contains many active ingredients such as steroidal saponins, protopanaxadiol, and protopanaxatriol, which are collectively referred to as ginsenosides [13]. Ginsenosides exert a wide range of pharmacological and therapeutic effects against oxidative stress, cancer, diabetes, vasorelaxation, and inflammation [13,14]. Ginsenosides exert beneficial effects on immunity, vitality, and libido [15,16]. Among ginsenosides, Rb1, Rb2, Rd, Rg1, Rg3, and Rh2 have been demonstrated to positively affect muscle strengthening by increasing mitochondrial biogenesis, inhibiting muscle degradation, and enhancing myoblast proliferation and myotube differentiation [17,18,19,20,21]. Ginsenoside Rc (gRc) has been reported to alleviate myocardial ischemic damage through its antioxidant and anti-inflammatory actions [22] and to improve cellular insulin resistance by increasing angiotensin-converting enzyme 2 expression [23]. However, its pharmacological efficacy and mechanism of action in muscle damage have not yet been reported.

In the present study, we investigated the effects of gRc on the hydrogen peroxide (H_2_O_2_)-induced inhibition of C2C12 myoblasts and degradation of C2C12 myotubes. Moreover, we examined whether gRc restores mitochondrial damage caused by oxidative stress and confirmed its mechanism of action through transcriptome analysis.

## 2. Materials and Methods

### 2.1. C2C12 Cell Culture and Differentiation to Myotubes

Murine skeletal muscle cell C2C12 myoblasts (CRL-1772; ATCC, Manassas, VA, USA) were cultured in a growth medium (GM, Dulbecco’s Modified Eagle Medium (DMEM) with 4.5 g/L glucose containing 10% heat-inactivated fetal bovine serum (FBS) and 100 IU penicillin/100 μg/mL streptomycin (P/S). To differentiate into myotubes, C2C12 myoblasts were grown in GM to reach more than 90% confluence, and the GM was removed and replaced with differentiation medium (DM, DMEM containing 2% heat-inactivated horse serum (HS) and P/S) every 2 days for 5–7 days. The cells were cultured at 37 °C in a humidified 5% CO_2_ incubator. DMEM, HS, and P/S were all purchased from Thermo Fisher Scientific (Waltham, MA, USA).

### 2.2. Reagents and Antibodies

gRc (PHL89210, ≥90% purity), H_2_O_2_ solution (216763), 4′,6-diamidino-2-phenylindole dihydrochloride (DAPI, D8417, ≥98% purity), and dimethyl sulfoxide (DMSO, D8418) were purchased from Sigma-Aldrich (St. Louis, MO, USA). gRc was dissolved in 100% DMSO to a final concentration of 20 mM and aliquots were stored at −20 °C. Anti-myosin heavy chain antibody (MyHC, MAB4470) was obtained from R&D Systems (Minneapolis, MN, USA). Antibodies against myogenic differentiation 1 (MyoD, sc-32758), muscle ring-finger protein-1 (MuRF1, sc-398608), and β-actin (sc-47778) were purchased from Santa Cruz Biotechnology (Santa Cruz, CA, USA). Anti-peroxisome proliferator-activated receptor-gamma co-activator 1α (PGC-1α, ab54481) and muscle atrophy F-box (MAFbx/Atrogin1, ab168371) antibodies were obtained from Abcam (Cambridge, MA, USA). Anti-nuclear respiratory factor 1 (NRF1, #46743), anti-Parkin (#4211), anti-α-tubulin (#2144), horseradish peroxidase (HRP)-conjugated anti-mouse IgG (#7076), and anti-rabbit IgG (#7074) were obtained from Cell Signaling Technology (Beverly, MA, USA).

### 2.3. Cytotoxicity Assay in Myoblasts and Myotubes

To determine cytotoxicity in myoblasts, cells (5 × 10^3^/well) were seeded in 96-well culture plates, cultured overnight, and treated with gRc, H_2_O_2_, or vehicle (0.1% DMSO) for 24 h. After removing the culture supernatants, the cells were washed twice with phosphate-buffered saline (PBS, Thermo Fisher Scientific), and viable cells were measured using the EZ-Cytox Enhanced Cell Viability Assay Kit (Daeil Lab Service Co., Ltd., Seoul, Republic of Korea) and a SpectraMax3 microplate reader (Molecular Devices, LLC, Sunnyvale, CA, USA) according to the manufacturer’s instructions. To determine cytotoxicity in myotubes, cells differentiated for 5 days (DD5) were treated with gRc, H_2_O_2_, or vehicle for 24 h, washed twice with PBS, and stained with crystal violet solution (0.2% crystal violet and 20% methanol) for 30 min at 25 °C. After washing completely with distilled water (DW), stained cells were dissolved with 1% sodium dodecyl sulfate (SDS) at 37 °C for 30 min and absorbance at 590 nm was detected using a SpectraMax3 microplate reader. To investigate the effects of gRc on H_2_O_2_ stimulation, the cells were pretreated with gRc for 12 h and then treated with H_2_O_2_ for 24 h.

### 2.4. Mouse PGC-1α (mPGC-1α) Luciferase Assay and β-Galactosidase Activity Assay

C2C12 myoblasts or myotubes (DD5) were seeded in 12-well culture plates at a density of 3 × 10^5^/well, incubated overnight, and transiently transfected with mPGC-1α-luciferase reporter plasmid (a kind gift from Professor Gyu-Un Bae, Sookmyung Women’s University, Republic of Korea) and β-galactosidase plasmid using the Mirus TransIT-X2 Dynamic Delivery System (Mirus Bio LLC, Madison, WI, USA) according to the manufacturer’s instructions. After 4 h, cells were treated with gRc, H_2_O_2_, or vehicle for 24 h and then lysed in 100 μL of Passive Lysis Buffer (Promega Co., Madison, WI, USA). Luciferase activity was determined using a Bio-GloTM Luciferase Assay Kit (Promega) and a SpectraMax L Luminometer (Molecular Devices) according to the manufacturer’s protocol. β-galactosidase activity in each sample was measured using O-nitrophenyl β-galactopyranoside (ONPG, Sigma-Aldrich) as the substrate and a SpectraMax3 microplate reader. β-galactosidase was used to correct the variability of transfection efficiency; therefore, the relative mPGC-1α luciferase activities were calculated after normalization to β-galactosidase activity.

### 2.5. Immunoblotting Analysis

To obtain total protein, cells were lysed using M-PER mammalian protein extraction reagent (Thermo Fisher Scientific), left at 4 °C for 30 min, and then centrifuged at 16,000× *g* for 15 min at 4 °C. After the clear supernatant was transferred to a new tube, it was subjected to a bicinchoninic acid assay to determine the protein concentration. Equal amounts of proteins (20 μg) were separated on SDS-polyacrylamide gel electrophoresis and transferred to an ImmobilonR-P PVDF membrane (Millipore, Bedford, MA, USA). After blocking with an EzBlock Chemi Solution (ATTO Korea, Daejeon, Republic of Korea) for 1 h at 25 °C, the membrane was incubated with a target-specific primary antibody (1:1000 dilution in blocking buffer) overnight at 4 °C. After washing with TBS-T solution (0.1% Tween in Tris-buffered saline), the membrane was incubated with an HRP-conjugated secondary antibody (1:4000 diluted in blocking buffer) for 1 h at 25 °C and then washed with TBS-T solution. Target proteins were detected using SuperSignal West Femto Maximum Sensitivity Substrate (Thermo Fisher Scientific) and ImageQuant LAS4000 mini (GE Healthcare, Piscataway, NJ, USA). Protein levels were measured using ImageJ software version 1.53t (National Institute of Health, Bethesda, MD, USA), and the relative band intensities of representative immunoblots from the two experiments were calculated after normalization to the value of β-actin or α-tubulin. Uncropped blot images were presented in Supplementary Figure S1.

### 2.6. Quantitation of Mitochondria Density

The DD5 myotubes were pretreated with gRc for 12 h and then treated with H_2_O_2_ for 24 h. After washing with PBS, the cells were incubated at 37 °C for 30 min in 50 nM MitoTracker Deep Red FM (M22426; Thermo Fisher Scientific). Red fluorescence was observed under a fluorescence microscope (Olympus TH4-200, Olympus Optical Co., Tokyo, Japan), and fluorescence density was analyzed using ImageJ software.

### 2.7. Immunocytochemistry for Myosin Heavy Chain (MyHC)

The DD5 myotubes differentiated on glass bottom dishes (SPL Life Sciences, Pocheon, Republic of Korea) were pretreated with gRc for 12 h and then treated with 0.25 mM H_2_O_2_ for 24 h. Cells were washed with PBS, fixed in 10% formalin solution, permeabilized in 0.1% Triton X-100, and blocked with 3% bovine serum albumin (Thermo Fisher Scientific) in PBS for 30 min at 25 °C at each step. After washing with PBS, the cells were stained with anti-MyHC antibody (1:1000 dilution in blocking buffer) overnight at 4 °C, followed by staining with Alexa Fluor 488-conjugated goat anti-mouse IgG antibody (1:1000 dilution in blocking buffer, Thermo Fisher Scientific) at 25 °C for 3 h. After nuclear counterstaining with DAPI, fluorescent images were captured under a fluorescence microscope. Using ImageJ software, the fusion index and myotube length were analyzed in seven representative images per group. The fusion index was calculated using the following formula: number of nuclei in multinucleated cells/number of total nuclei ×100.

### 2.8. Measurement of Intracellular ATP Content

The DD5 myotubes were pretreated with gRc for 12 h and then treated with 0.25 mM H_2_O_2_ for 24 h. Intracellular ATP content was determined using the ATP Bioluminescent Assay Kit (FLAA, Sigma-Aldrich) according to the manufacturer’s protocol. Briefly, the cells were lysed with DW and centrifuged at 13,000× *g* for 10 min at 4 °C to collect the clear cell lysates. ATP standards or cell lysates were mixed with an ATP assay mix solution and luminescence was measured immediately using a SpectraMax L luminometer. Intracellular ATP levels were calculated from an ATP calibration curve and relative values were obtained after normalization to the amount of protein used in the assay.

### 2.9. Detection of Oxidative Stress

To detect ROS and mitochondrial superoxide in live cells, CellROX™ Green and MitoSOX™ Red reagents were used, respectively. In brief, myoblasts or myotubes at DD5 grown on glass bottom dishes were pretreated with gRc for 12 h and then exposed to 0.25 mM H_2_O_2_ for 6 h. After washing the cells with Hanks’ Balanced Salt Solution (HBSS, Thermo Fisher Scientific), they were incubated in 5 μM CellROX™ Green reagent or MitoSOX™ Red reagent for 15 min at 37 °C. After washing with HBSS, fluorescent images of cells were captured using a fluorescence microscope.

### 2.10. RNA Sequencing Data Acquisition and Preprocessing

Total RNA (1 µg) was processed to prepare the mRNA sequencing library following the manufacturer’s instructions provided with the MGIEasy RNA Directional Library Prep kit (#1000006386; MGI Tech, Shenzhen, China). The constructed library was quantified using a QauntiFluor^®^ ssDNA System (E3190; Promega). Subsequently, the prepared DNA nanoballs were sequenced on the MGISeq platform (MGI Tech) with 100 bp paired-end reads. FastQC (v0.11.9) was used to assess the read quality. Common sections of the MGISEQ adapter sequences were eliminated using TrimGalore (v0.6.5). The resulting trimmed reads were mapped to the GRCm38 (mm10) mouse reference genome using STAR (v2.7.3a) [24] with default configurations. To quantify gene expression levels, we used RSEM (v1.3.3) [25] along with the GRCm38.86 gene annotation to obtain the expected read counts and transcript per million (TPM) values.

### 2.11. Functional Enrichment Analysis

Functional enrichment analysis using RNA-seq data was conducted using R software (v4.2.1). Differential gene expression analysis was performed between the different groups (e.g., H_2_O_2_ vs. vehicle or gRc + H_2_O_2_ vs. H_2_O_2_) using the DESeq2 package (v1.36). This analysis generated a ranked gene list based on the Wald statistics. To identify the pathways or functions that were overrepresented among the genes at the top or bottom of the ranked gene list, we used the Gene Set Enrichment Analysis (GSEA) method implemented in the fgsea package (v1.22) [26]. Functional annotation of genes was obtained from the Molecular Signature Database (MSigDB) on 20 January 2023, using the msigdbr package (v7.5.1). We only used curated gene sets sourced from reliable databases within MSigDB, including Hallmark, Gene Ontology (GO), Reactome, and WikiPathways. GSEA produced enriched functional terms with normalized enrichment scores (NESs) and *p*-values, which indicated the strength and significance of the association with the gene sets.

### 2.12. Statistical Analysis

Data were analyzed using GraphPad Prism 9 software (GraphPad Software, San Diego, CA, USA). Values are expressed as the mean ± standard error of the mean (SEM) of multiple experiments. The differences in means among groups were analyzed by one-way ANOVA (Dunnett’s multiple comparison test), and a value of *p* < 0.05 was considered statistically significant.

## 3. Results

### 3.1. gRc Reduces H_2_O_2_-Induced Cytotoxicity in C2C12 Myoblasts

**Figure 1:**
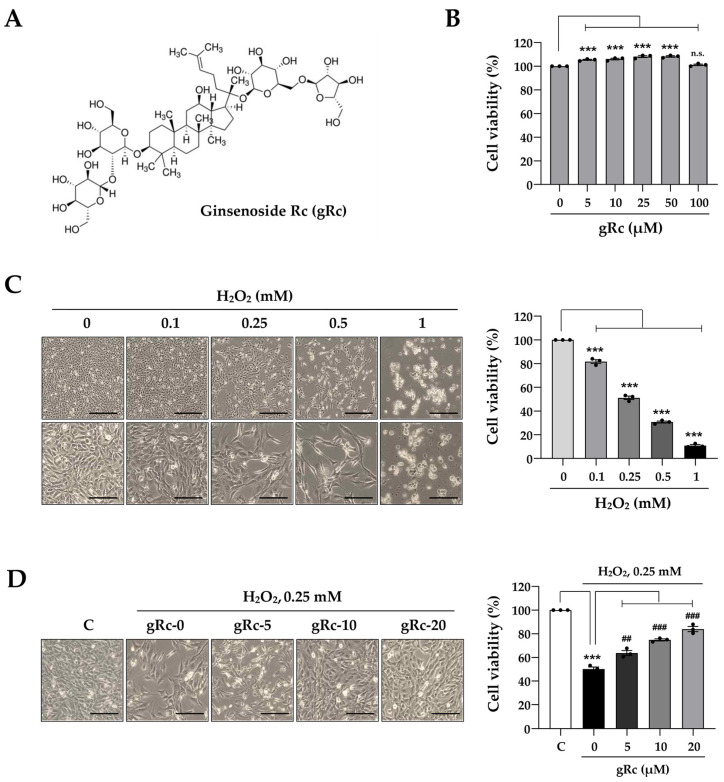
Effects of gRc on the cell viability of H_2_O_2_-treated myoblasts. (**A**) The chemical structure of ginsenoside Rc (gRc). (**B**) Myoblasts were treated with gRc up to 100 μM for 24 h and cell viability was measured. (**C**) Myoblasts were treated with H_2_O_2_ for 24 h. Morphological changes were observed under an inverted microscope and cell viability was measured. Scale bar = 100 μm. (**D**) Myoblasts were pretreated with gRc for 12 h, and then further incubated with H_2_O_2_ for 24 h. Relative cell viability compared to vehicle-treated control cells was expressed as the mean ± SEM (*n* = 3). Dots are individual values. *** *p* < 0.001 vs. vehicle-treated cells, ^##^ *p* < 0.01 and ^###^ *p* < 0.001 vs. H_2_O_2_ + vehicle-treated cells; n.s., non-significant.

To evaluate the beneficial effects of gRc on muscle cells, we assessed the viability of myoblasts after treatment with gRc for 24 h. The chemical structure of gRc was presented in Figure 1A. As shown in Figure 1B, gRc did not exhibit any cytotoxic effects on myoblasts at concentrations up to 100 μM. Interestingly, at concentrations ranging from 5 μM to 50 μM, gRc slightly enhanced the cell viability by approximately 5% to 7%. As previously reported [27], H_2_O_2_ treatment at 0.1, 0.25, 0.5, and 1 mM decreased myoblast viability by approximately 17.6%, 47.8%, 67.9%, and 89.7%, respectively (Figure 1C). In the present study, 0.25 mM H_2_O_2_ was selected to induce oxidative stress and the highest concentration of gRc to examine the protected effects was set at 20 μM. To determine the protective effects of gRc on H_2_O_2_-induced cytotoxicity, myoblasts were pretreated with gRc for 12 h and then further incubated with 0.25 mM H_2_O_2_ for 24 h. As shown in Figure 1D, gRc markedly inhibited the H_2_O_2_-induced decrease in myoblast viability in a dose-dependent manner. Particularly, gRc at 20 μM maintained cell viability at approximately 95% of that of the control cells.

### 3.2. gRc Decreases H_2_O_2_-Induced Oxidative Stress and Increases Mitochondrial Functions in C2C12 Myoblasts

**Figure 2:**
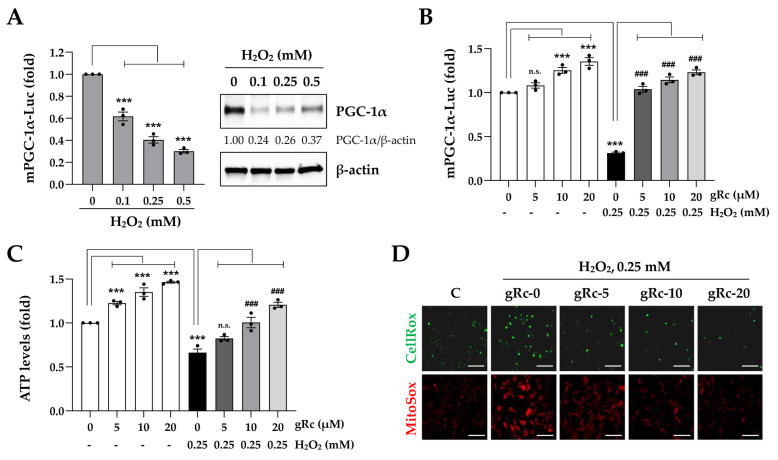
Effects of gRc on the mitochondrial biogenesis and oxidative stress in H_2_O_2_-treated myoblasts. (**A**) Myoblasts were co-transfected with mPGC-1α-Luc and β-galactosidase plasmids. After treatment with indicated concentrations of H_2_O_2_ for 24 h, luciferase activity was measured. The PGC-1α protein levels were determined by Western blotting. (**B**) After co-transfection with mPGC-1α-Luc and β-galactosidase plasmids, myoblasts were treated with gRc alone or in combination with H_2_O_2_. After normalization to β-galactosidase activity, relative mPGC-1α promoter activity was expressed as the mean ± SEM (*n* = 3). Dots are individual values. (**C**) After treatment of myoblasts with gRc alone or in combination with H_2_O_2_, ATP level was determined and presented as the mean ± SEM (*n* = 3). Dots are individual values. (**D**) Myoblasts were pretreated with gRc and then exposed to H_2_O_2_ for 6 h. Cellular ROS and mitochondrial superoxide generation was observed under a fluorescence microscope. Scale bar = 100 μm. *** *p* < 0.001 vs. vehicle-treated cells, ^###^ *p* < 0.01 vs. H_2_O_2_ + vehicle-treated cells; n.s., non-significant.

PGC-1α, the master transcriptional co-activator, has been reported to enhance muscle mitochondrial biogenesis and function and to be associated with the regulation of cellular redox balance and attenuation of H_2_O_2_-induced apoptotic cell death [28,29,30]. Because oxidative stress is a negative regulator of PGC-1α, an increase in PGC-1α expression and activity is crucial for counteracting oxidative stress-induced muscle damage. In line with previous reports, we confirmed that H_2_O_2_ dramatically reduced PGC-1α promoter activity and PGC-1α protein expression in myoblasts (Figure 2A). gRc at 10 and 20 μM significantly increased PGC-1α promoter activity in myoblasts approximately 1.2-fold and 1.4-fold, respectively. Furthermore, gRc pretreatment effectively prevented the H_2_O_2_-induced reduction in PGC-1α activity to higher levels compared to those in the H_2_O_2_-untreated control cells (Figure 2B). Treatment with 0.25 mM H_2_O_2_ led to approximately 30% ATP deprivation in myoblasts. In contrast, gRc treatment substantially increased intracellular ATP synthesis, irrespective of the presence of H_2_O_2_, and gRc pretreatment effectively mitigated the decrease in ATP synthesis caused by H_2_O_2_ (Figure 2C). To detect intracellular ROS and mitochondrial superoxide levels in myoblasts, we labeled the cells with CellRox green and MitoSox red dyes, respectively. Following a 6 h exposure to H_2_O_2_, myoblasts exhibited intense green and red fluorescence, indicative of increased ROS and mitochondrial superoxide production. In contrast, pretreatment with gRc significantly decreased both the green and red fluorescence intensities in a dose-dependent manner (Figure 2D). Collectively, these findings indicate that gRc effectively alleviates oxidative stress and enhances mitochondrial function by increasing PGC-1α activity, thereby protecting myoblasts against oxidative stress-induced cytotoxicity.

### 3.3. gRc Inhibits H_2_O_2_-Induced C2C12 Myotube Degradation

**Figure 3:**
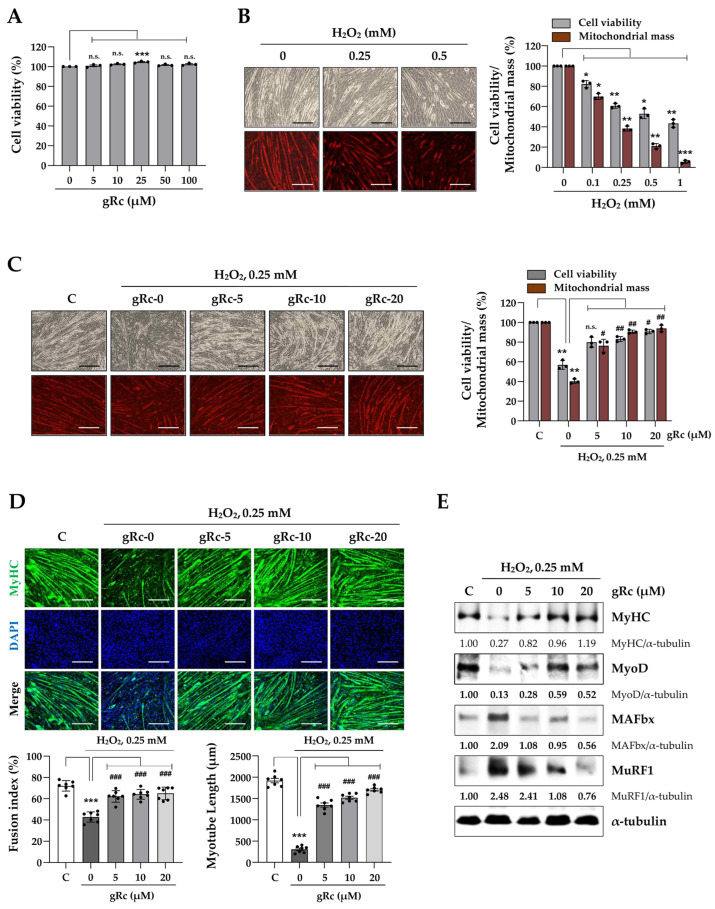
Effects of gRc on H_2_O_2_-induced degradation of myotubes. (**A**) C2C12 myotubes were treated with gRc for 24 h and cell viability was measured. (**B**) Myotubes were treated with H_2_O_2_ up to 1 mM for 24 h. Relative cell viability and mitochondrial mass compared to vehicle-treated cells were expressed as the mean ± SEM (*n* = 3). Dots are individual values. (**C**) Myotubes were pretreated with gRc for 12 h and further incubated with H_2_O_2_. After 24 h, cell viability and mitochondrial mass were determined and relative values were expressed as the mean ± SEM (*n* = 3). Dots are individual values. (**D**) Myotubes were pretreated with gRc for 12 h and then treated with H_2_O_2_. After 24 h, cells were subjected to immunofluorescence staining for MyHC (green) and DAPI (blue). Fusion index and myotube length were quantitated and presented as the mean ± SEM (*n* = 7). Dots are individual values. (**E**) Effects of gRc on the expression of MyHC, MyoD, MAFbx, and MuRF1 in H_2_O_2_-treated myotubes were determined by Western blotting. * *p* < 0.05, ** *p* < 0.01, and *** *p* < 0.001 vs. vehicle-treated cells, ^#^ *p* < 0.05, ^##^ *p* < 0.01, ^###^ *p* < 0.001 vs. H_2_O_2_ + vehicle-treated cells; n.s., non-significant. Scale bar = 100 μm.

To evaluate the protective effects of gRc against H_2_O_2_-induced muscle degradation, we first examined the effects of gRc and H_2_O_2_ treatment on C2C12 myotubes. gRc treatment up to 100 μM did not induce cytotoxicity; conversely, the treatment slightly increased cell viability (Figure 3A). In contrast, H_2_O_2_ disrupted cellular morphology and decreased cell viability and mitochondrial mass in myotubes (Figure 3B). Pretreatment with gRc prior to H_2_O_2_ stimulation effectively recovered both cell viability and mitochondrial mass of myotubes to levels comparable to those of the control cells (Figure 3C). Immunofluorescence staining showed that the myotubes expressed substantial levels of MyHC, a muscle differentiation marker, with a fusion index of approximately 70%. H_2_O_2_ treatment significantly reduced MyHC expression, the fusion index by 38.4%, and myotube length by 81.6% compared to control myotubes. However, pretreatment with gRc maintained MyHC expression at a level similar to that in the control cells, even under H_2_O_2_ stimulation. In addition, gRc pretreatment significantly prevented the decline in the fusion index and myotube length induced by H_2_O_2_ (Figure 3D). Western blot analysis revealed that H_2_O_2_ treatment dramatically reduced the protein levels of MyHC and myogenic MyoD in myotubes, while simultaneously increasing the levels of muscle-degrading proteins, including MAFbx and MuRF1. Pretreatment with gRc significantly inhibited the H_2_O_2_-induced downregulation of MyHC and MyoD and upregulation of MAFbx and MuRF1 (Figure 3E). Overall, these findings indicate that gRc efficiently suppressed H_2_O_2_-induced myotube degradation.

### 3.4. gRc Enhances Mitochondrial Biogenesis and Reduces Oxidative Stress in C2C12 Myotubes under H_2_O_2_ Treatment

**Figure 4:**
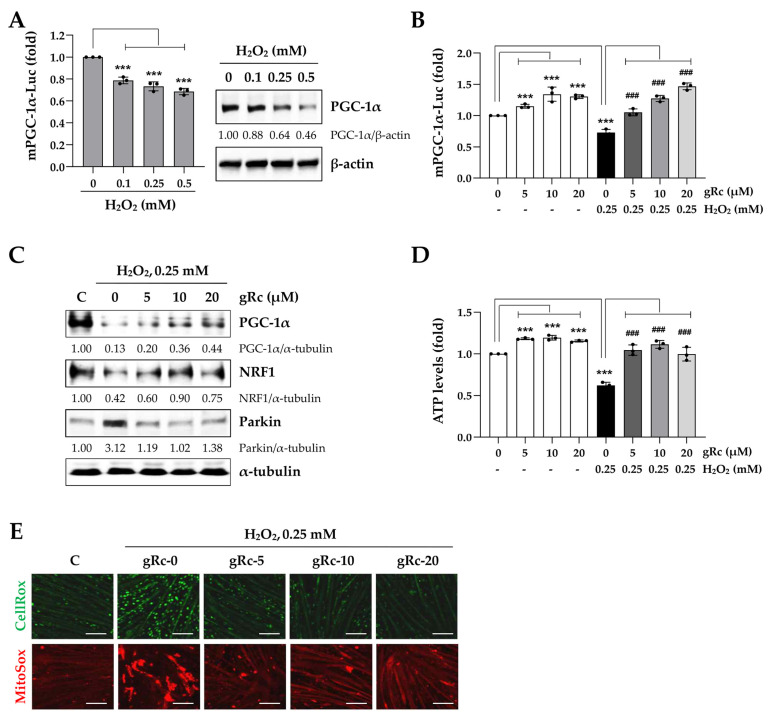
Effects of gRc on the mitochondrial biogenesis and oxidative stress in H_2_O_2_-treated myotubes. (**A**) mPGC-1α promoter activity and PGC-1α protein level in myotubes were measured after treatment with H_2_O_2_. (**B**) mPGC-1α promoter activity in myotubes was measured after treatment with gRc alone or in combination with H_2_O_2_. Relative mPGC-1α activity was expressed as the mean ± SEM (*n* = 3). Dots are individual values. (**C**) The protein levels of PGC-1α, NRF1, and Parkin in H_2_O_2_-treated myotubes were measured by Western blotting. (**D**) After treatment with gRc alone or in combination with H_2_O_2_, ATP level was determined and presented as the mean ± SEM (*n* = 3). Dots are individual values. (**E**) Myotubes were pretreated with gRc and further incubated in the presence of H_2_O_2_. After 6 h, cellular ROS (green) and mitochondrial superoxide (red) were observed under a fluorescence microscope. *** *p* < 0.001 vs. vehicle-treated cells, ^###^ *p* < 0.001 vs. H_2_O_2_ + vehicle-treated cells. Scale bar = 100 μm.

Consistent with the findings in myoblasts (Figure 2A), H_2_O_2_ treatment markedly reduced the promoter activity of PGC-1α as well as its protein expression in myotubes (Figure 4A). gRc considerably increased the PGC-1α promoter activity in the myotubes and gRc pretreatment prior to H_2_O_2_ stimulation almost completely blocked the reduction in PGC-1α activity (Figure 4B). PGC-1α and its downstream nuclear transcription factor, NRF-1/2, are crucial to the maintenance of mitochondrial function, biogenesis, and energy metabolism under oxidative stress [31,32,33]. As shown in Figure 4C, Western blot analysis revealed that gRc pretreatment significantly recovered the protein expression of PGC-1α and NRF1, which was reduced by H_2_O_2_. In addition, we observed that H_2_O_2_ increased the protein expression of Parkin, a mitochondrial stress marker, whereas gRc pretreatment efficiently suppressed this H_2_O_2_-induced increase in Parkin expression. Upon treatment with H_2_O_2_, intracellular ATP levels in the myotubes were significantly reduced to approximately 50%. In contrast, gRc slightly increased the ATP levels in myotubes and effectively prevented the H_2_O_2_-induced decrease in ATP levels (Figure 4D). Although H_2_O_2_ strongly induced the production of intracellular ROS (green fluorescence) and mitochondrial superoxide (red fluorescence) in the myotubes, their production was almost completely suppressed by gRc pretreatment (Figure 4E). Overall, these results suggest that gRc exerts a protective effect against muscle damage caused by oxidative stress by regulating mitochondrial biogenesis and increasing PGC-1α activity.

### 3.5. gRc-Induced Transcriptome Reveals the Upregulation of Mitochondrial Biogenesis and Muscle-Protective Pathways against Oxidative Stress

**Figure 5:**
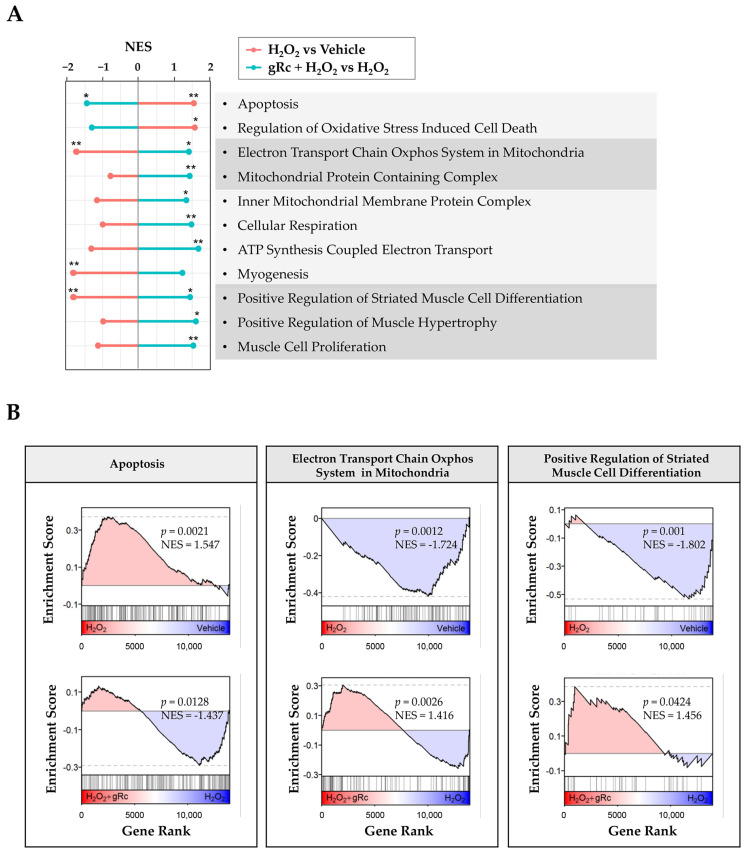
Transcriptome analysis of the effect of gRc in H_2_O_2_-treated C2C12 myotubes. (**A**) Normalized enrichment score (NES) of the gene set enrichment analysis (GSEA) results of H_2_O_2_ treatment compared to vehicle and the H_2_O_2_ + gRc treatment compared to the H_2_O_2_ treatment. The pathway terms were sourced from Gene Ontology—Biological Process (“Regulation of Oxidative Stress Induced Cell Death”, “Positive Regulation of Striated Muscle Cell Differentiation”, “ATP Synthesis Coupled Electron Transport”, “Muscle Cell Proliferation, Cellular Respiration”, and “Positive Regulation of Muscle Hypertrophy”), Gene Ontology—Cellular Component (“Mitochondrial Protein Containing Complex” and “Inner Mitochondrial Membrane Protein Complex”), Reactome (“Apoptosis” and “Myogenesis”), and WikiPathways (“Electron Transport Chain Oxphos System in Mitochondria”). * *p* < 0.05, ** *p* < 0.01. (**B**) The selected GSEA plots of H_2_O_2_ treatment compared to vehicle (top) and the H_2_O_2_ + gRc treatment compared to the H_2_O_2_ treatment (bottom).

To further elucidate the mechanisms underlying the protective effects of gRc against oxidative stress, we performed RNA-seq analysis of C2C12 myotubes treated with H_2_O_2_ and gRc. H_2_O_2_ treatment significantly upregulated the expression of genes associated with oxidative stress-induced cell death and apoptosis (Figure 5A,B). However, pretreatment with gRc prior to H_2_O_2_ treatment resulted in the downregulation of these genes, indicating its potential to mitigate cell death caused by H_2_O_2_-induced oxidative stress. Remarkably, gRc pretreatment significantly upregulated the expression of genes involved in mitochondrial biogenesis, including those associated with the mitochondrial protein complex, electron transport chain of the oxidative phosphorylation system in mitochondria, and ATP synthesis (Figure 5A,B). In contrast, these genes were downregulated by H_2_O_2_ treatment alone. Moreover, gRc pretreatment enhanced pathways related to myogenesis and muscle cell proliferation/differentiation, which were reduced by H_2_O_2_ (Figure 5A,B). Collectively, these results provide strong evidence supporting the effective mitigation of oxidative stress-induced muscle damage by gRc through the regulation of mitochondrial biogenesis and facilitation of muscle proliferation in skeletal muscle cells.

## 4. Discussion

Excessive production of ROS is known as oxidative stress. Oxidative stress results from an imbalance between the production and accumulation of ROS and protective antioxidant systems of the body. Superoxide anion (·O_2_−) and hydroxyl radical (·OH) are highly unstable species with unpaired electrons and are the most active free radicals capable of initiating oxidation and producing further ROS, including H_2_O_2_, hypochlorous acid (HOCl), and peroxynitrite (ONOO−). Adequate ROS levels play a key physiological role as mediators of intracellular signal transduction. However, when produced in excess, ROS have a direct harmful effect on biological structures such as proteins, lipids, and nucleic acids, resulting in severe damage to cells and tissues. Endogenous ROS are mainly by-products of oxygen metabolism, whereas exogenous ROS are triggered by various environmental stresses, including radiation, drugs, smoking, heavy metals, and chronic inflammation [11,12,33].

Mitochondria contribute to the ATP synthesis and biosynthesis of nucleic acids, lipids, amino acids, purines, and steroids. Furthermore, they control intracellular Ca^2+^ homeostasis, and are involved in cell division and programmed cell death [9]. Free radicals and superoxide anions are produced in the mitochondria during oxidative metabolism and mitochondrial respiration. When oxidative stress caused by ROS generation overwhelms the mitochondrial antioxidant defense system, it causes mitochondrial DNA damage and mitochondrial dysfunction, which in turn causes biomolecular and cellular injury, cell death, and inflammation, and may contribute to the progression of various diseases, including neurodegenerative and cardiovascular diseases. Therefore, oxidative stress has emerged as a potent therapeutic target for disease control [7,9,10].

In healthy adults, skeletal muscle accounts for 40–50% of the total body mass, and a decrease in skeletal muscle mass decreases the quality of life and increases morbidity and mortality. Under conditions such as wasting diseases (e.g., cancer, diabetes, and sepsis), the administration of several drugs (e.g., cancer chemotherapy, glucocorticoids, and statins), aging, and prolonged immobilization (e.g., fractures and surgical interventions), skeletal muscle loss proceeds through a multifactorial process [5]. Mitochondrial dysfunction is a common denominator of skeletal muscle loss. Therefore, studies on promoting muscle differentiation and function and suppressing muscle loss by enhancing mitochondrial function have been conducted. The fruit extract of *Lycium chinense* and its bioactive compound, betaine, were found to significantly increase the expression of mitochondrial biogenesis-regulating factors such as PGC-1α, Sirt-1, NRF-1, and TFAM. They also enhanced the differentiation of C2C12 cells and increased ATP content and glucose uptake in C2C12 myotubes [34]. Schisandrin A, an active dibenzocyclooctadiene lignan derived from the fruits of *Schisandra chinensis*, effectively blocked H_2_O_2_-induced ROS accumulation, attenuated DNA damage, and prevented apoptotic cell death in C2C12 myoblasts by preserving mitochondrial function [35]. In addition, various herbal extracts (e.g., *Prunus mume* fruit extract, *Moringa oleifera* leaf extract, and loquat leaf extract), traditional herbal medicines (e.g., Jakyakgamcho-Tang), and phytochemicals (e.g., morin and honokiol) exhibit beneficial effects in alleviating muscle damage under oxidative stress [27,36,37,38,39].

Ginseng root (*Panax ginseng* Meyer) has long been used as an ethnomedicinal herb. Pharmacological studies have demonstrated that ginseng possesses diverse bioactive properties, including anti-stress, anti-inflammatory, anti-cancer, anti-aging, and anti-diabetic effects [13,16]. Ginseng contains several bioactive components including ginsenosides. To date, more than a hundred ginsenosides have been extracted from the various parts of ginseng. Among them, the major glycosylated ginsenosides, such as Rb1, Rb2, Rc, Rd, Re, and Rg1, are the most abundant, constituting more than 80% of the total ginsenosides. Several studies have demonstrated the efficacy of ginsenosides for improving mitochondrial function, inhibiting muscle loss, and enhancing muscle function. Rg1 was found to promote myogenic differentiation in C2C12 cells by activating the promyogenic kinases Akt and p38 MAPK. Additionally, Rg1 effectively prevented dexamethasone-induced myotube degradation by activating the Akt/mTOR pathway. Rb1 and Rb2 have also been reported to upregulate myotube growth and myogenic differentiation, suggesting that they may prevent and treat age-related muscle atrophy [17,18,19,20,22,23,40,41]. Unlike these ginsenosides, in our study, gRc did not promote myogenic differentiation in regard to myotube formation and MyHC expression. Meanwhile, Rc suppressed t-BHP-induced ROS generation and directly scavenged superoxide free radicals in HEK293 cells [42]. In addition, Rc protected HaCaT keratinocytes against UVB-induced photooxidative damage [43].

Owing to the absence of studies on the beneficial effects of Rc on muscle cells focusing on its antioxidant efficacy, we investigated the protective effects of gRc on oxidative stress-induced mitochondrial damage, the inhibition of myoblast growth, and myotube degradation for the first time. In C2C12 myoblasts, gRc effectively suppressed H_2_O_2_-induced cytotoxicity and at 20 μM of gRc, cell viability was maintained at a level similar to that of the control cells (Figure 1). Moreover, gRc dramatically suppressed the H_2_O_2_-induced generation of intracellular ROS as well as mitochondrial superoxide, restored PGC-1α promoter activity, and increased ATP synthesis (Figure 2). Consistent with our data, in normal and ischemia/reperfusion-injured cardiac and neuronal models, gRc promoted energy metabolism by activating the SIRT1 and PGC-1α pathway and reduced mitochondrial oxidative stress and apoptosis [44,45]. Considering the primary localization of PGC-1α in the nucleus and large structure of gRc, we speculate that the activation of the PGC-1α pathway by gRc is plausibly a consequence of its antioxidant effects rather than a direct interaction between PGC-1α and gRc. These results reinforce the protective effect of gRc against oxidative stress through the potentiation of mitochondrial biogenesis. In C2C12 myotubes, we also found that gRc exerted muscle-preserving effects by maintaining mitochondrial mass and biogenesis, inhibiting muscle proteolysis, and scavenging intracellular ROS and mitochondrial superoxide (Figure 3 and Figure 4). Furthermore, transcriptome analysis of C2C12 myotubes confirmed the protective role of gRc against H_2_O_2_-induced oxidative stress via these pathways in skeletal muscle (Figure 5). One notable drawback of our study is the lack of in vivo studies. Although our in vitro investigations have provided useful information about gRc, they cannot fully represent the complex biological systems found in living animals. Furthermore, although we examined the overall effect of gRc on muscle atrophy, no extensive and thorough investigation of the essential molecular mechanisms and complicated molecular-level interactions was conducted. Therefore, to overcome these limitations, additional research using animal models of muscle atrophy and more comprehensive transcriptome analyses are required.

## 5. Conclusions

Our findings provide pharmacological evidence of gRc to protect muscle cells against oxidative stress and enhance mitochondrial biogenesis, accompanied by the activation of the PGC-1α pathway. In a follow-up study, we will investigate the efficacy of gRc under various in vitro and in vivo muscle loss conditions (e.g., cancer cachexia, drug-induced muscle atrophy, and age-related sarcopenia) to verify whether gRc may be a potential remedy for muscle loss and muscle weakness. In addition, we plan to identify and verify critical molecular mechanisms regulated by gRc at in vitro and in vivo levels through further studies.

## Data Availability

The raw sequencing data (FASTQ files) and processed count data were uploaded to the Gene Expression Omnibus under the accession number GSE198653.

## Supplementary Materials

The following supporting information can be downloaded at: https://www.mdpi.com/article/10.3390/antiox12081576/s1, Figure S1: Full blot images of Western blot analysis.

## References

[1] Cho M.R., Lee S., Song S.K. (2022). A Review of Sarcopenia Pathophysiology, Diagnosis, Treatment and Future Direction. J. Korean Med. Sci..

[2] Marcell T.J. (2003). Sarcopenia: Causes, consequences, and preventions. J. Gerontol. A Biol. Sci. Med. Sci..

[3] Xia L., Zhao R., Wan Q., Wu Y., Zhou Y., Wang Y., Cui Y., Shen X., Wu X. (2020). Sarcopenia and adverse health-related outcomes: An umbrella review of meta-analyses of observational studies. Cancer Med..

[4] Larsson L., Degens H., Li M., Salviati L., Lee Y.I., Thompson W., Kirkland J.L., Sandri M. (2019). Sarcopenia: Aging-Related Loss of Muscle Mass and Function. Physiol. Rev..

[5] Jang J.Y., Kim D., Kim N.D. (2023). Pathogenesis, Intervention, and Current Status of Drug Development for Sarcopenia: A Review. Biomedicines.

[6] Hyatt H., Deminice R., Yoshihara T., Powers S.K. (2019). Mitochondrial dysfunction induces muscle atrophy during prolonged inactivity: A review of the causes and effects. Arch. Biochem. Biophys..

[7] Hyatt H.W., Powers S.K. (2021). Mitochondrial Dysfunction Is a Common Denominator Linking Skeletal Muscle Wasting Due to Disease, Aging, and Prolonged Inactivity. Antioxidants.

[8] Sena L.A., Chandel N.S. (2012). Physiological roles of mitochondrial reactive oxygen species. Mol. Cell.

[9] Bhatti J.S., Bhatti G.K., Reddy P.H. (2017). Mitochondrial dysfunction and oxidative stress in metabolic disorders—A step towards mitochondria based therapeutic strategies. Biochim. Biophys. Acta (BBA)—Mol. Basis Dis..

[10] Chen M.M., Li Y., Deng S.L., Zhao Y., Lian Z.X., Yu K. (2022). Mitochondrial Function and Reactive Oxygen/Nitrogen Species in Skeletal Muscle. Front. Cell Dev. Biol..

[11] Lian D., Chen M.M., Wu H., Deng S., Hu X. (2022). The Role of Oxidative Stress in Skeletal Muscle Myogenesis and Muscle Disease. Antioxidants.

[12] Chen M., Wang Y., Deng S., Lian Z., Yu K. (2022). Skeletal muscle oxidative stress and inflammation in aging: Focus on antioxidant and anti-inflammatory therapy. Front. Cell Dev. Biol..

[13] Leung K.W., Wong A.S. (2010). Pharmacology of ginsenosides: A literature review. Chin. Med..

[14] Lee C.H., Kim J.H. (2014). A review on the medicinal potentials of ginseng and ginsenosides on cardiovascular diseases. J. Ginseng Res..

[15] Huang Q., Gao S., Zhao D., Li X. (2021). Review of ginsenosides targeting mitochondrial function to treat multiple disorders: Current status and perspectives. J. Ginseng Res..

[16] Ratan Z.A., Haidere M.F., Hong Y.H., Park S.H., Lee J.O., Lee J., Cho J.Y. (2021). Pharmacological potential of ginseng and its major component ginsenosides. J. Ginseng Res..

[17] Dong W., Chen W., Zou H., Shen Z., Yu D., Chen W., Jiang H., Yan X., Yu Z. (2022). Ginsenoside Rb1 Prevents Oxidative Stress-Induced Apoptosis and Mitochondrial Dysfunction in Muscle Stem Cells via NF-kappaB Pathway. Oxid. Med. Cell. Longev..

[18] Lim W.C., Shin E.J., Lim T.G., Choi J.W., Song N.E., Hong H.D., Cho C.W., Rhee Y.K. (2022). Ginsenoside Rf Enhances Exercise Endurance by Stimulating Myoblast Differentiation and Mitochondrial Biogenesis in C2C12 Myotubes and ICR Mice. Foods.

[19] Kim R., Kim J.W., Lee S.J., Bae G.U. (2022). Ginsenoside Rg3 protects glucocorticoid-induced muscle atrophy in vitro through improving mitochondrial biogenesis and myotube growth. Mol. Med. Rep..

[20] Li F., Li X., Peng X., Sun L., Jia S., Wang P., Ma S., Zhao H., Yu Q., Huo H. (2017). Ginsenoside Rg1 prevents starvation-induced muscle protein degradation via regulation of AKT/mTOR/FoxO signaling in C2C12 myotubes. Exp. Ther. Med..

[21] Kim M.J., Koo Y.D., Kim M., Lim S., Park Y.J., Chung S.S., Jang H.C., Park K.S. (2016). Rg3 Improves Mitochondrial Function and the Expression of Key Genes Involved in Mitochondrial Biogenesis in C2C12 Myotubes. Diabetes Metab. J..

[22] Shi L., Fu W., Xu H., Li S., Yang X., Yang W., Sui D., Wang Q. (2022). Ginsenoside Rc attenuates myocardial ischaemic injury through antioxidative and anti-inflammatory effects. Pharm. Biol..

[23] Wang Y., Fu W., Xue Y., Lu Z., Li Y., Yu P., Yu X., Xu H., Sui D. (2021). Ginsenoside Rc Ameliorates Endothelial Insulin Resistance via Upregulation of Angiotensin-Converting Enzyme 2. Front. Pharmacol..

[24] Dobin A., Davis C.A., Schlesinger F., Drenkow J., Zaleski C., Jha S., Batut P., Chaisson M., Gingeras T.R. (2013). STAR: Ultrafast universal RNA-seq aligner. Bioinformatics.

[25] Li B., Dewey C.N. (2011). RSEM: Accurate transcript quantification from RNA-Seq data with or without a reference genome. BMC Bioinform..

[26] Subramanian A., Tamayo P., Mootha V.K., Mukherjee S., Ebert B.L., Gillette M.A., Paulovich A., Pomeroy S.L., Golub T.R., Lander E.S. (2005). Gene set enrichment analysis: A knowledge-based approach for interpreting genome-wide expression profiles. Proc. Natl. Acad. Sci. USA.

[27] Kim Y.S., Yuk H.J., Kim D.S. (2021). Effect of Jakyakgamcho-Tang Extracts on H_2_O_2_-Induced C2C12 Myoblasts. Molecules.

[28] Choi H.I., Kim H.J., Park J.S., Kim I.J., Bae E.H., Ma S.K., Kim S.W. (2017). PGC-1alpha attenuates hydrogen peroxide-induced apoptotic cell death by upregulating Nrf-2 via GSK3beta inactivation mediated by activated p38 in HK-2 Cells. Sci. Rep..

[29] Chen L., Qin Y., Liu B., Gao M., Li A., Li X., Gong G. (2022). PGC-1alpha-Mediated Mitochondrial Quality Control: Molecular Mechanisms and Implications for Heart Failure. Front. Cell Dev. Biol..

[30] Gill J.F., Delezie J., Santos G., McGuirk S., Schnyder S., Frank S., Rausch M., St-Pierre J., Handschin C. (2019). Peroxisome proliferator-activated receptor gamma coactivator 1alpha regulates mitochondrial calcium homeostasis, sarcoplasmic reticulum stress, and cell death to mitigate skeletal muscle aging. Aging Cell.

[31] Abu Shelbayeh O., Arroum T., Morris S., Busch K.B. (2023). PGC-1alpha Is a Master Regulator of Mitochondrial Lifecycle and ROS Stress Response. Antioxidants.

[32] Scarpulla R.C. (2011). Metabolic control of mitochondrial biogenesis through the PGC-1 family regulatory network. Biochim. Biophys. Acta.

[33] Baar K., Song Z., Semenkovich C.F., Jones T.E., Han D.H., Nolte L.A., Ojuka E.O., Chen M., Holloszy J.O. (2003). Skeletal muscle overexpression of nuclear respiratory factor 1 increases glucose transport capacity. FASEB J..

[34] Ma J., Meng X., Kang S.Y., Zhang J., Jung H.W., Park Y.K. (2019). Regulatory effects of the fruit extract of *Lycium chinense* and its active compound, betaine, on muscle differentiation and mitochondrial biogenesis in C2C12 cells. Biomed. Pharmacother..

[35] Choi Y.H. (2018). Schisandrin A prevents oxidative stress-induced DNA damage and apoptosis by attenuating ROS generation in C2C12 cells. Biomed. Pharmacother..

[36] Ceci R., Maldini M., Olson M.E., Crognale D., Horner K., Dimauro I., Sabatini S., Duranti G. (2022). *Moringa oleifera* Leaf Extract Protects C2C12 Myotubes against H_2_O_2_-Induced Oxidative Stress. Antioxidants.

[37] Kwon Y.H., Jang J.Y., Lee J.H., Choi Y.W., Choi Y.H., Kim N.D. (2023). Loquat leaf extract inhibits oxidative stress-induced DNA damage and apoptosis via AMPK and Nrf2/HO-1 signaling pathways in C2C12 cells. Appl. Sci..

[38] Park C., Choi S.H., Jeong J.W., Han M.H., Lee H., Hong S.H., Kim G.Y., Moon S.K., Kim W.J., Choi Y.H. (2020). Honokiol ameliorates oxidative stress-induced DNA damage and apoptosis of c2c12 myoblasts by ROS generation and mitochondrial pathway. Anim. Cells Syst..

[39] Lee M.H., Han M.H., Lee D.S., Park C., Hong S.H., Kim G.Y., Hong S.H., Song K.S., Choi I.W., Cha H.J. (2017). Morin exerts cytoprotective effects against oxidative stress in C2C12 myoblasts via the upregulation of Nrf2-dependent HO-1 expression and the activation of the ERK pathway. Int. J. Mol. Med..

[40] Go G.Y., Lee S.J., Jo A., Lee J., Seo D.W., Kang J.S., Kim S.K., Kim S.N., Kim Y.K., Bae G.U. (2017). Ginsenoside Rg1 from *Panax ginseng* enhances myoblast differentiation and myotube growth. J. Ginseng Res..

[41] Go G.Y., Jo A., Seo D.W., Kim W.Y., Kim Y.K., So E.Y., Chen Q., Kang J.S., Bae G.U., Lee S.J. (2020). Ginsenoside Rb1 and Rb2 upregulate Akt/mTOR signaling-mediated muscular hypertrophy and myoblast differentiation. J. Ginseng Res..

[42] Kim D.H., Park C.H., Park D., Choi Y.J., Park M.H., Chung K.W., Kim S.R., Lee J.S., Chung H.Y. (2014). Ginsenoside Rc modulates Akt/FoxO1 pathways and suppresses oxidative stress. Arch. Pharm. Res..

[43] Oh Y., Lim H.W., Park K.H., Huang Y.H., Yoon J.Y., Kim K., Lim C.J. (2017). Ginsenoside Rc protects against UVB-induced photooxidative damage in epidermal keratinocytes. Mol. Med. Rep..

[44] Huang Q., Su H., Qi B., Wang Y., Yan K., Wang X., Li X., Zhao D. (2021). A SIRT1 Activator, Ginsenoside Rc, Promotes Energy Metabolism in Cardiomyocytes and Neurons. J. Am. Chem. Soc..

[45] Xue Y., Fu W., Yu P., Li Y., Yu X., Xu H., Sui D. (2023). Ginsenoside Rc Alleviates Myocardial Ischemia-Reperfusion Injury by Reducing Mitochondrial Oxidative Stress and Apoptosis: Role of SIRT1 Activation. J. Agric. Food Chem..

