# Supplementary material for: Ginsenoside Rc, an Active Component of Panax ginseng, Alleviates Oxidative Stress-Induced Muscle Atrophy via Improvement of Mitochondrial Biogenesis
Source: Antioxidants (Basel). 2023 Aug 7;12(8):1576. doi: 10.3390/antiox12081576 (PMC10451796; doi:10.3390/antiox12081576)
Supplement: Supplementary file 1 [file antioxidants-12-01576-s001.zip › antioxidants-2516842-supplementary.pdf]

Supplementary Materials for

Ginsenoside Rc, an active component of *Panax ginseng*, alleviates oxidative stress-induced muscle atrophy via improvement of mitochondrial biogenesis

Aeyung Kim, Sang-Min Park, No Soo Kim, and Haeseung Lee

*Corresponding Author: Aeyung Kim (aykim71@kiom.re.kr), Haeseung Lee (haeseung@pusan.ac.kr)

This PDF file includes: Figure S1

Figure S1

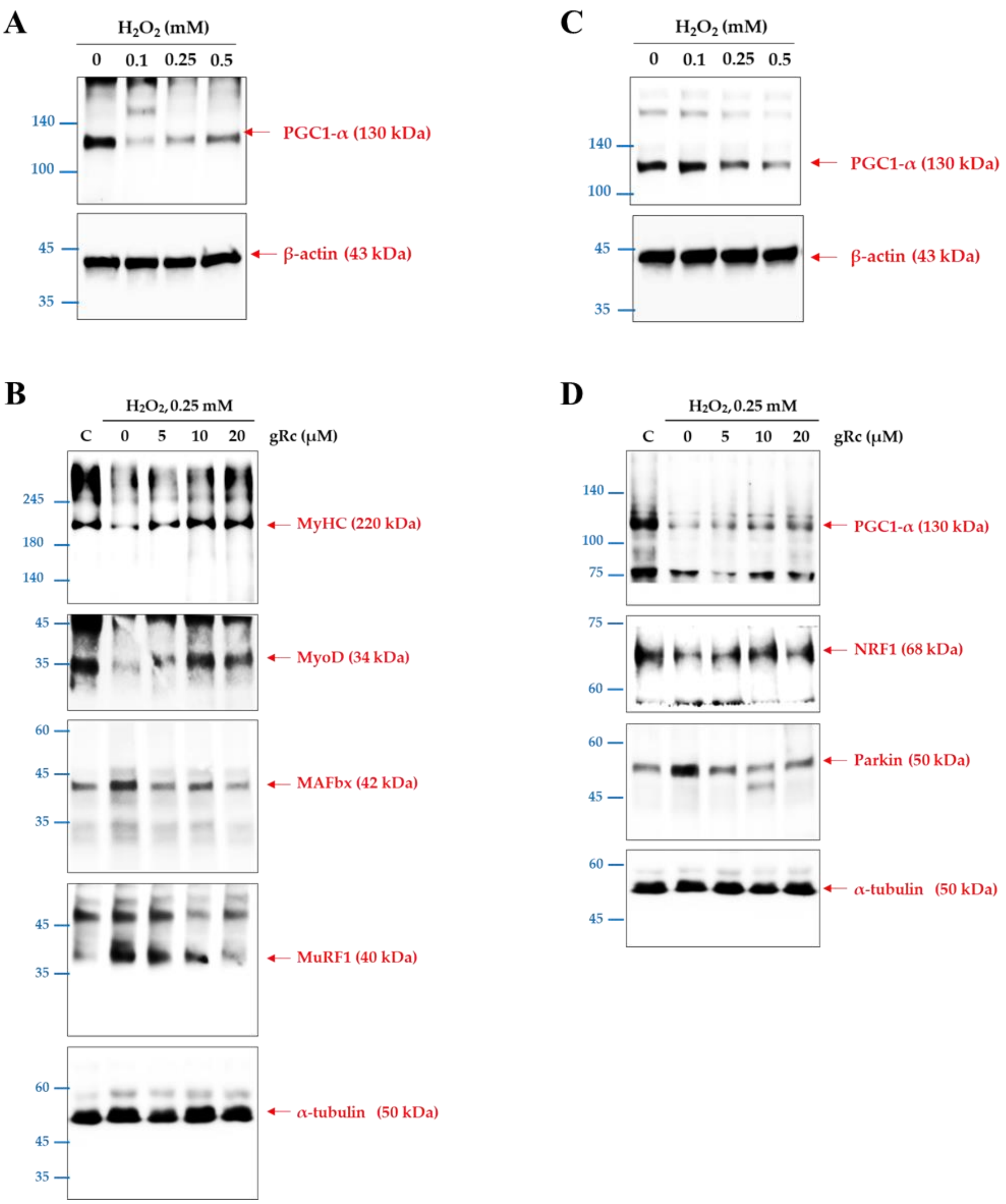

Figure S1. Uncropped western blot images corresponding to Figure 2A **(A)**, Figure 3E **(B)**, Figure 4A **(C)**, and Figure 4C **(D)**.
